# Identification of Genes Encoding CENP-A and Heterochromatin Protein 1 of *Lipomyces starkeyi* and Functional Analysis Using *Schizosaccharomyces pombe*

**DOI:** 10.3390/genes11070769

**Published:** 2020-07-08

**Authors:** Yuko Takayama

**Affiliations:** 1 Department of Biosciences, School of Science and Engineering, Teikyo University, 1-1 Toyosatodai, Utsunomiya, Tochigi 320–8551, Japan; takayama@nasu.bio.teikyo-u.ac.jp; Tel.: +81-28-627-7242; 2 Division of Integrated Science and Engineering, Graduate School of Science and Engineering, Teikyo University Graduate Schools, 1–1 Toyosatodai, Utsunomiya, Tochigi 320–8551, Japan

**Keywords:** centromere, CENP-A, heterochromatin, oleaginous yeast

## Abstract

Centromeres function as a platform for the assembly of multiple kinetochore proteins and are essential for chromosome segregation. An active centromere is characterized by the presence of a centromere-specific histone H3 variant, CENP-A. Faithful centromeric localization of CENP-A is supported by heterochromatin in almost all eukaryotes; however, heterochromatin proteins have been lost in most Saccharomycotina. Here, identification of CENP-A (CENP-A^L.s.^) and heterochromatin protein 1 (Lsw1) in a Saccharomycotina species, the oleaginous yeast *Lipomyces starkeyi,* is reported. To determine if these proteins are functional, the proteins in *S. pombe*, a species widely used to study centromeres, were ectopically expressed. CENP-A^L.s.^ localizes to centromeres and can be replaced with *S. pombe* CENP-A, indicating that CENP-A^L.s.^ is a functional centromere-specific protein. Lsw1 binds at heterochromatin regions, and chromatin binding is dependent on methylation of histone H3 at lysine 9. In other species, self-interaction of heterochromatin protein 1 is thought to cause folding of chromatin, triggering transcription repression and heterochromatin formation. Consistent with this, it was found that Lsw1 can self-interact. *L. starkeyi* chromatin contains the methylation of histone H3 at lysine 9. These results indicated that *L. starkeyi* has a primitive heterochromatin structure and is an attractive model for analysis of centromere heterochromatin evolution.

## 1. Introduction

The histone H3 variant, CENP-A, is a highly conserved protein. In most organisms, CENP-A is a hallmark of a functional centromere, a specific locus of a chromosome that organizes the assembly of the kinetochore proteins. The conserved essential function of centromeres has been preserved despite changes in DNA sequence, length, and organization as species evolve [1]. The centromere type comprises two categories by its length: point and regional. All studied species of the Saccharomycotina have point centromeres [2,3]. In *Saccharomyces cerevisiae*, a specific 125 bp DNA sequence is necessary and sufficient to define a centromere. Most eukaryotes have ‘regional centromeres’, which consist of large blocks of repetitive DNA sequences [1]. The regional centromeres in the fission yeast *Schizosaccharomyces pombe* consist of two domains: a 10–15 kb central domain (*cnt* and *imr*) flanked by a 10–60 kb heterochromatic outer (*otr*) domain composed of *dg* and *dh* elements [4,5]. The heterochromatic *otr* domain contains methylated histone H3 at lysine 9 (H3K9me), which is characteristic of silent heterochromatin. The *otr* region is a target of the RNA interference (RNAi) machinery, and RNAi activity is required for heterochromatin formation. Functional RNAi machinery includes Dicer, Argonaute, and RNA-dependent RNA polymerase [6,7].

Heterochromatin protein 1 (HP1) is a chromatin-associated protein responsible for the propagation and maintenance of chromatin structures, and HP1 orthologs are evolutionarily conserved in most eukaryotes. Moreover, multiple HP1 paralogs can be found in humans, mice (HP1α, HP1β, HP1γ) and *Drosophila* (HP1a, HP1b, HP1c, HP1d, HP1e). Two paralogs (Swi6 and Chp2) related to HP1 are present in fission yeast; however, *S. cerevisiae* lacks identifiable homologs of HP1. These heterochromatin proteins have two characteristic domains: a chromo domain (CD) and a chromoshadow domain (CSD). The CD binds H3K9me and the CSD facilitates homodimerization of HP1 and binds other chromatin proteins involved in heterochromatin assembly, resulting in transcriptional repression. These domains are connected by an unstructured hinge region [8]. The hinge region has been implicated in sequence-independent RNA and DNA binding [8,9]. Fission yeast has a single member of the SUV39 histone methyltransferase family protein, Clr4, which methylates histone H3K9 [10]. In contrast, almost all Saccharomycotina lost the genes encoding Swi6 and Clr4, which are required for making heterochromatin via histone H3K9 methylation [2,11]. Orthologs of these genes were found in the genome sequence of the oleaginous yeast *Lipomyces starkeyi*, which is a Saccharomycotina species [2]. At present, however, it is not clear whether these ortholog genes are functional.

*L. starkeyi* is a powerful lipid producer with great industrial potential as it is a useful host for efficient lipid production. Most studies of *L. starkeyi* have focused on obtaining a strain that accumulates more lipids than the control strain. Despite this industrial relevance, limited information has been reported on biological processes in this species, especially the cell cycle and chromosomes. In this study, orthologs of centromere protein CENP-A (CENP-A^L.s.^) and heterochromatin protein 1 (Lsw1) were identified and cloned. These genes were expressed in fission yeast and analyzed for molecular function. CENP-A^L.s.^ displays functionality as it can rescue the fission yeast Cnp1 (CENP-A homolog) thermosensitive mutant *cnp1-1* and binds to the centromere core region. Lsw1 is localized specifically to the heterochromatin regions, and its localization is dependent on H3K9 methylation. *L. starkeyi* chromatin contains H3K9me, suggesting that *L. starkeyi* preserved a primitive heterochromatin structure that relies on H3K9me, even though that structure was lost in other Saccharomycotina. These findings might be helpful in understanding centromere heterochromatin evolution.

*L. starkeyi* yeast is a remarkable organism with regard to biofuel production. There are many studies on medium components or culture conditions that support the most efficient accumulation of biofuel in the cells. Development of a gene transfer method using a centromeric plasmid would help these efforts. In the future, if the sequence of the *L. starkeyi* centromere can be revealed by Chromatin immunoprecipitation (ChIP)-sequencing using immunoprecipitation of CENP-A^L.s.^, then a centromeric plasmid specific to *L. starkeyi* can be constructed.

## 2. Materials and Methods

### 2.1. Media and Strains

Fission yeast media have been previously described [12,13]. The fission yeast strains used in this study are listed in Table 1.

### 2.2. Cloning of CENP-A^L.s.^ and Lsw1

*L. starkeyi* NBRC1289 cells were grown in 30 mL of YPDA medium (1% yeast extract, 2% peptone, 2% D-glucose and 50 mg/L adenine), and total RNA was prepared using the acid-phenol method [13,15]. *L. starkeyi* cells were harvested by centrifugation, the cell pellet was suspended in 720 μL of TES (10 mM Tris-HCl pH 7.5, 10 mM EDTA, 0.5% SDS), and 720 μL of acidic phenol-chloroform was added (pH 4.5, Nacalai Tesque, Inc., Kyoto, Japan). After vigorous mixing, the cell mixture was incubated for 1 h at 65 °C. After centrifugation, the aqueous layer was re-extracted with acidic phenol-chloroform. The aqueous layer was then treated with 720 μL of chloroform. After centrifugation, the aqueous layer was precipitated with cold ethanol. The total RNA pellet was then rinsed with 70% ethanol and dissolved in distilled water. Total RNA was used for the synthesis of cDNA using ReverTra Ace qPCR RT Master Mix with gDNA remover (TOYOBO, Osaka, Japan). The coding sequences of CENP-A^L.s.^ and Lsw1 were amplified by Prime STAR (TaKaRa, Shiga, Japan) using the synthesized cDNAs as a template. PCR primer sequences are listed in Table 2. The PCR products were cloned into the pGEM-T vector by TA-cloning (Promega, Madison, WI, USA). The inserted DNA fragments were then sequenced and ligated into the pRep41 or Rep81-GFP plasmid (pGP4110 or pGP8110, [16]). The pRep42-Lsw1-Flag plasmid was constructed using an In-Fusion HD cloning kit (Clontech, Mountain View, CA, USA). The pRep42N plasmid was digested with *Sal*I and *Not*I. The Lsw1 fragment was amplified with Lsw1-F and Lsw1-R primers using pGP4110-Lsw1 as a template. The Flag fragment was amplified with Flag-F and Flag-R primers using pFA6a-3flag as a template. PCR-amplified fragments were separated by agarose gel electrophoresis and purified using the Wizard SV gel and PCR clean-up system (Promega). The purified fragments (Lsw1 and Flag) and digested pRep42 were then incubated with In-fusion HD enzyme premix for 15 min at 50 °C. The incubated solution was transformed into DH5α competent cells (Nippon Gene Co., Toyama, Japan), and the final constructed plasmid was verified by nucleotide sequencing.

### 2.3. S. pombe Transformation

*S. pombe* cells were transformed by electroporation using an ELEPO21 (Nepa Gene, Chiba, Japan). The cells were then grown to 2 × 10^7^ cells/mL, placed on ice for 15 min, and collected by centrifugation. The cell pellet was suspended in 0.1 M lithium acetate and incubated for 45 min at the appropriate culture temperature (i.e., 30 °C for HM123, YTP1847, YTP1889 cells and 26 °C for YTP1840, YTP1901 cells or 23 °C for Sp525 cells). Next, 1 M DTT was added to a final concentration of 10 mM, and the suspension was incubated for 15 min at the appropriate culture temperature. After centrifugation, the cell pellet was washed twice with ice-cold 1 M sorbitol. The final pellet was resuspended in ice-cold 1 M sorbitol at a volume that results in a solution containing 1 × 10^9^ cells/mL. The cell suspension was mixed with 10 ng plasmid and then transferred to an ice-chilled cuvette with a 2 mm gap. The electroporation conditions were as follows: poring pulse—1500 V, pulse length 3.5 ms, pulse interval 50 ms, two pulses, polarity +; transfer pulse—100 V, pulse length 50 ms, pulse interval 50 ms, three pulses, polarity +/−. The resistance value was adjusted to approximately 45–20 kΩ just before electroporation. After electroporation, the cells were immediately added to ice-cold 1 M sorbitol and plated on selection medium.

### 2.4. Microscopy

Microscopy was performed as described in [20]. The cells used in HM123 cells containing pGP8110-Cnp1 or pGP4110-CENP-A^L.s.^ and YTP1889 cells, YTP1847 cells containing pGP4110 or pGP4110-Lsw1, and YTP1840 cells containing pGP4110-Lsw1 were cultured and fixed in methanol at −80 °C, then washed three times with PBS. Next, the fixed cells were mixed with 200 ng/mL DAPI. For observation, a DM5500B digital microscope equipped with a 100× objective lens (N.A. 1.30) and DFC 310FX digital CCD color camera (Leica Microsystems GmbH, Wetzlar, Germany) was used.

### 2.5. Chromatin Immunoprecipitation (ChIP) Assay

The ChIP assay was performed as described in [21]. The cells used in Sp525 cells containing pGP8110-Cnp1 or pGP4110-CENP-A^L.s.^ and YTP1889 and YTP1847 or YTP1901 containing pGP4110-Lsw1 were cultured in EMM2 medium with thiamine and appropriate supplements and washed three times with distilled water. The washed cells were then cultured in EMM2 with appropriate supplements without thiamine and fixed with 3% formaldehyde. The fixed cells were broken with glass beads, and the cell lysates were sonicated. The sonicated lysates were incubated with anti-GFP antibody (Roche, 1814460) for 2 h at 4 °C followed by incubation with Dynabeads α-Mouse IgG (VERITAS, Tokyo, Japan) overnight at 4 °C. The beads were washed and then incubated in TE with 1% SDS overnight at 65 °C. The sample was then treated with proteinase-K, and the DNA was extracted with phenol-chloroform and precipitated with ethanol. The DNA was analyzed by real-time PCR using a 7500 fast system (Life Technologies, Waltham, MA, USA) and Gene Ace SYBER qPCR Mix low ROX (Nippon Gene Co.). Primer sequences are listed in Table 2. For each sample, the amount of immunoprecipitated DNA was divided by the amount of DNA in the corresponding whole-cell extract sample.

### 2.6. Co-Immunoprecipitation

Co-immunoprecipitation was performed as described in [22]. Δ*swi6* cells (YTP1847) were co-transformed with pRep42-Lsw1-3Flag and either pGP4110 or pGP4110-Lsw1. The pGP4110 and pRep42 plasmid markers are *LEU2* or *ura4^+^*, respectively. Plasmid-containing cells were cultured in EMM2 without thiamine for 23 h at 30 °C. The cell pellets were suspended in lysis buffer (50 mM HEPES pH7.5, 1 mM EDTA, 200 mM NaCl, 1 mM β-mercaptoethanol, 10% glycerol, 0.5% Triton X-100), and cell lysates were prepared using a FastPrep-24 system (MP-Biomedicals, Irvine, CA, USA) with glass beads. The cell lysates were then incubated with anti-GFP antibody (Roche, Mannheim, Germany, 1814460) for 2 h at 4 °C followed by addition of Dynabeads α-Mouse IgG (VERITAS) for 3 h at 4 °C. The beads were washed with lysis buffer and analyzed by Western blotting using anti-GFP (Roche, 1814460) and anti-M2 (SIGMA, St. Louis, MO, USA, F3165-0.2MG).

### 2.7. Western Blotting Analysis

Cell lysates were prepared using the alkali-trichloroacetic acid method [13,23]. The antibodies used were anti-H3 (Abcam, Cambridge, UK, ab-1791), anti-H3K9me2 (Abcam, ab-115159), anti-H3K4me2 (Millipore, Burlington, MA, USA, 07–030), and anti-PSTAIR (Sigma-Aldrich, St. Louis, MO, USA, P7962).

## 3. Results

### 3.1. Centromere-Specific Histone H3 Variant of L. starkeyi

#### 3.1.1. Identification of *L. starkeyi* CENP-A

**Figure 1:**
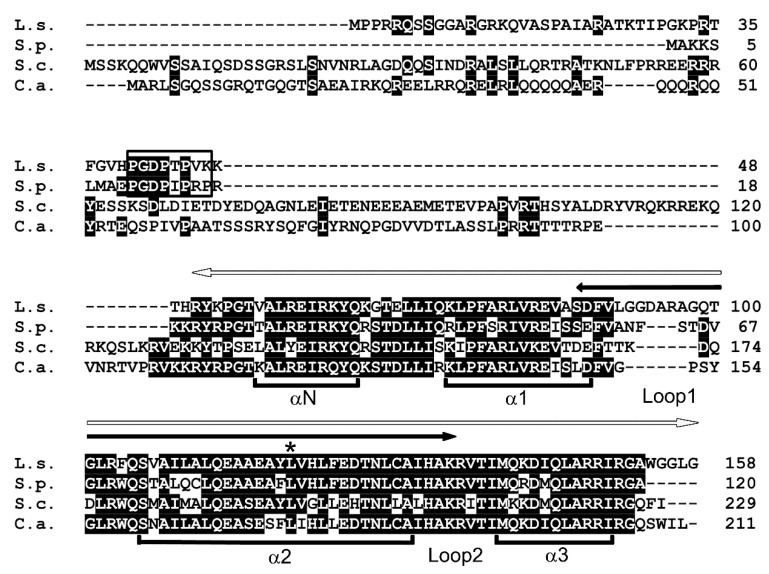
Amino acid sequence alignment of CENP-A proteins. *L. starkeyi* (L.s.), *S. pombe* (S.p.), *S. cerevisiae* (S.c.), and *C. albicans* (C.a.). Black boxes indicate identical amino acid residues. White arrow, conserved histone fold domain. Black arrow, centromere targeting domain. Open box, genomic stability-regulating site within CENP-A N-terminus (GRANT) motif. Asterisk, the position of the *cnp1-1* mutation. The α-helices are indicated (αN, α1, α2, and α3).

CENP-A is a centromere-specific histone H3 variant and a highly conserved protein. The *Saccharomyces cerevisiae* CENP-A homolog Cse4 is the most studied CENP-A in a Saccharomycotina species. Thus, in an attempt to identify a CENP-A homolog in *L. starkeyi*, which is also among the Saccharomycotina, Cse4 was used as a query in a BLAST search of *L. starkeyi*. In all organisms, CENP-A proteins share a highly conserved C-terminal histone H3-like region (histone fold domain), and BLAST analysis with Cse4 retrieved a histone H3 sequence. However, this search did not identify a candidate *L. starkeyi* CENP-A homolog; thus, a similar search was next performed using a fission yeast CENP-A homolog, *Schizosaccharomyces pombe* Cnp1. A gene (LIPSTDRAFT 73903) that encodes a protein of 158 amino acids was identified and named CENP-A^L.s.^ (Figure 1). Multiple alignment showed that the conserved C-terminal histone fold domain of CENP-A^L.s.^ was 74%, 67%, and 80% identical to *S. pombe* Cnp1, *S. cerevisiae* Cse4, and *Candida albicans* CaCse4, respectively (Figure 1, white arrow). Loop I, the region important for centromeric DNA targeting [24], was observed as a variation among these species. Loop I of CENP-A^L.s.^ is 16 amino acids longer than those found in the other yeast CENP-As (*S. pombe*, 13 a.a.; *S. cerevisiae* and *C. albicans*, 10 a.a.).

CENP-A proteins have a highly conserved C-terminal histone fold domain and centromere targeting domain. The N-terminal domain, however, is diversified among CENP-A proteins from different organisms, and it is thought to epigenetically regulate centromeric identity [12,20,25,26]. Moreover, this region is also involved in a ubiquitination event that results in the degradation of mis-targeted CENP-A [27,28]. The N-terminal domain of CENP-A^L.s.^ consists of 50 amino acids, which is shorter than those found in other Saccharomycotina, i.e., in Cse4 (130 a.a.) or CaCse4 (110 a.a.). The length of the N-terminal domain of CENP-A^L.s.^ is significantly different from that of Cse4. This difference in the N-terminal domain provides an explanation for the finding that CENP-A^L.s.^ could not be identified when Cse4 was used as a query in BLAST search. In fission yeast, the proline-rich motif GRANT (genomic stability-regulating site within CENP-A N-terminus) is essential for centromeric targeting of Cnp1 [29]. A proline-rich GRANT motif was found in the N-terminal region of CENP-A^L.s.^ (Figure 1, open box). These findings suggest that CENP-A^L.s.^ is a centromere-specific histone H3 variant protein in *L. starkeyi*.

#### 3.1.2. CENP-A^L.s.^ Is Functionally Equivalent to Fission Yeast Cnp1

**Figure 2:**
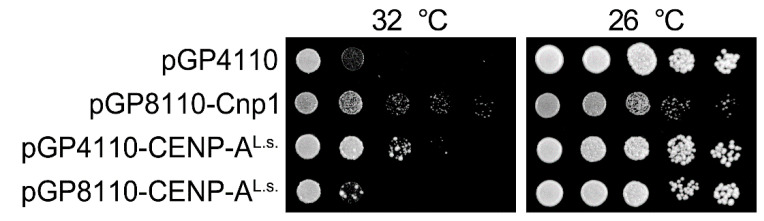
The ts phenotype of *cnp1-1* is suppressed by overexpression of CENP-A^L.s.^. Serial dilutions of *cnp1-1* cells (Sp525) containing pGP4110, pGP8110-Cnp1, pGP8110-CENP-A^L.s.^, or pGP4110-CENP-A^L.s.^ were spotted and grown on PMG medium in the absence of thiamine for 5 days at 26 °C or at 32 °C.

CENP-A localization at the centromere is essential for cell viability. Whether CENP-A^L.s.^ functions as a centromeric protein in *L. starkeyi* was next investigated. Several techniques for genetic modification of *L. starkeyi* have been reported. For example, gene deletion via insertion of an antibiotic resistance gene [30] and insertion of an exogenous gene [31,32,33] have been reported for *L. starkeyi*. Unfortunately, little is known about plasmids or genome integration-based epitope tag fusion systems in *L. starkeyi*. As an alternative approach, fission yeast was used. There are many useful molecular biology methods available for fission yeast, and they have been widely used to study the centromere. In addition, previous work has shown that fission yeast Cnp1 can be replaced with *S. cerevisiae* Cse4 [34]. First, to ascertain whether CENP-A^L.s.^ can replace Cnp1, the Cnp1 temperature-sensitive (ts) mutant cells, *cnp1-1*, were used. In the protein encoded by the *cnp1-1* allele, the 87th amino acid is exchanged from leucine to glutamine (L87Q, Figure 1, asterisk) and fails to form colonies in the temperature range 30 to 36 °C [12,14]. CENP-A^L.s.^ was isolated by RT-PCR using *L. starkeyi* cDNA, fused at the N-terminus with GFP, and placed under the control of the thiamine-controlled promoters *nmt41* or *nmt81* (resulting in the plasmids pGP4110 or pGP8110, respectively). These promoters are induced when thiamine is removed from the medium, and expression levels of genes under the control of the *nmt41* promoter are stronger than those under control of the *nmt81* promoter [16]. It was found that *cnp1-1* cells overexpressing GFP alone did not grow on medium lacking thiamine at restrictive temperature (Figure 2, pGP4110). In contrast, this ts phenotype of *cnp1-1* cells was rescued by expression of CENP-A^L.s.^ using the *nmt41* promoter (Figure 2, pGP4110-CENP-A^L.s.^). The observation that levels of CENP-A^L.s.^ expressed from the *nmt81* promoter were lower those from the *nmt41* promoter likely explains the finding that the ts phenotype of *cnp1-1* was not suppressed. This result indicates that CENP-A^L.s.^ rescues thermo sensitivity of *cnp1-1*.

**Figure 3:**
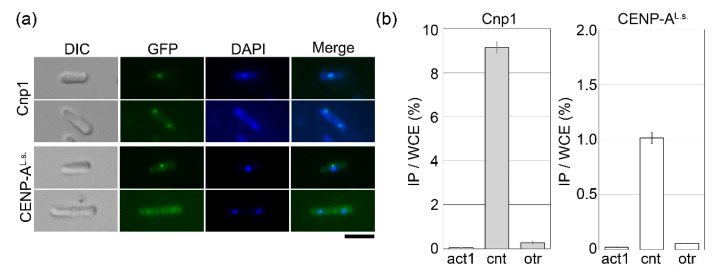
CENP-A^L.s.^ can localize to the fission yeast centromere. (**a**) Wild-type cells (HM123) containing pGP8110-Cnp1 (Cnp1) or pGP4110-CENP-A^L.s.^ (CENP-A^L.s.^) were grown on minimal medium in the absence of thiamine at 33 °C for 18 h, then fixed with methanol. Bar, 10 μm. (**b**) *cnp1-1* cells (Sp525) containing pGP8110-Cnp1 (Cnp1) or pGP4110-CENP-A^L.s.^ (CENP-A^L.s.^) were grown on minimal medium in the absence of thiamine at 30 °C for 25 h, then fixed with 3% formaldehyde. DNA co-immunoprecipitated using anti-GFP antibodies was quantified by real-time PCR using primers that recognize the inner centromere (*cnt*) or outer centromere (*otr*) region. *act1* was used as a negative control. The amount of immunoprecipitated DNA was calculated as a percentage of whole-cell extract (WCE). Error bars indicate the standard deviation from three independent experiments.

Next, to identify the subcellular localization of CENP-A^L.s.^, cells overexpressing GFP-tagged CENP-A^L.s.^ were observed by fluorescence microscopy. When CENP-A^L.s.^ was overexpressed in *cnp1-1* cells (Figure 2), the fluorescent signal was very weak. Therefore, wild-type cells were used instead of *cnp1-1* cells. In fission yeast, centromeres are clustered, and consistent with this, the Cnp1-GFP signal appears as a single dot in the nucleus (Figure 3a, Cnp1) [14]. For GFP-CENP-A^L.s.^, a single dot was clearly visible in cells with a single nucleus, and the GFP fusion protein was detected but very weak in cells with two nuclei (Figure 3a, CENP-A^L.s.^). The fission yeast centromere consists of central and outer regions [4,5]. Cnp1 accumulation is tightly restricted to the central region (*cnt*), whereas the outer region (*otr*) forms heterochromatin. To demonstrate whether the CENP-A^L.s.^ faithfully binds at the central region of the centromere, chromatin immunoprecipitation (ChIP) assay was employed. The *cnp1-1* cells overexpressing GFP-tagged Cnp1 or CENP-A^L.s.^ were fixed by formaldehyde and immunoprecipitated with an anti-GFP antibody. Cnp1 was found to bind to the central core (*cnt*) but not to the outer region (*otr*) of centromeres (Figure 3b, Cnp1), consistent with previous reports [20,26]. CENP-A^L.s.^ accumulation was detected specifically in the *cnt* central region and not in the *otr* heterochromatin region (Figure 3b, CENP-A^L.s.^). Taken together, these results show that CENP-A^L.s.^ functions as a centromere-specific histone H3 variant protein.

### 3.2. Heterochromatin Protein 1 of L. starkeyi

#### 3.2.1. Identification of *L. starkeyi* Heterochromatin Protein 1

**Figure 4:**
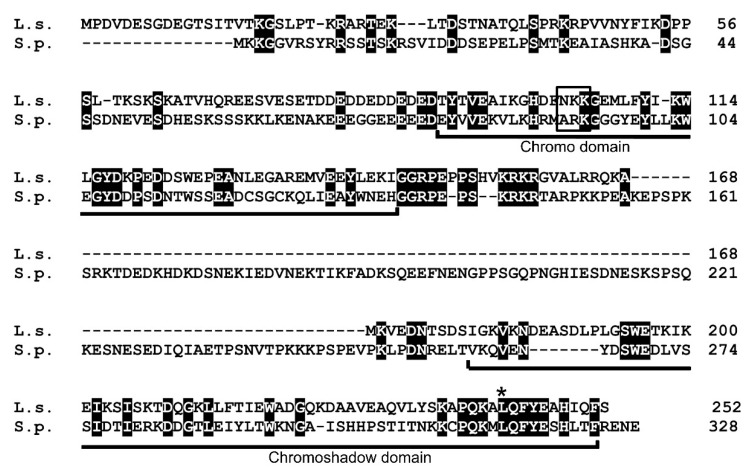
Amino acid sequence alignment of Lsw1 (L.s.) with Swi6 (S.p.). Black boxes indicate identical amino acid residues. The chromo domain (CD) and chromoshadow domain (CSD) regions are indicated. Open box, ARK-loop motif. Asterisk, leucine residue required for self-interaction of Swi6.

In fission yeast, pericentromeric heterochromatin regions (*otr*) are required for establishment of Cnp1 centromere localization [18,35] and are regulated by methylation of histone H3 at lysine 9 (H3K9me), which serves as a binding site for Swi6 [10]. The Swi6 protein has two conserved domains, the N-terminal chromo domain (CD) and C-terminal chromoshadow domain (CSD), separated by a hinge region. Although the sequence of the *L. starkeyi* centromere has not been revealed, a previous study reported [2] that *L. starkeyi* is the only sequenced Saccharomycotina species with orthologs of *S. pombe* Swi6. Then, heterochromatin protein 1 family Swi6 homolog of *L. starkeyi* was searched by BLAST. From this analysis, a gene (LIPSTDRAFT 112810) that encodes a 252 amino acid protein was identified. The protein sequence indicates the presence of the characteristic CD and CSD (Figure 4); thus, the gene that encodes this protein was named Lsw1 (for *L. starkeyi* Swi6 homolog 1). The hinge region has been implicated in sequence-independent DNA and RNA binding [8]. The hinge region of human HP1α is 43 amino acids, of *Drosophila* HP1 is 65 amino acids, and of fission yeast Swi6 is 124 amino acids [36]. The hinge region of Lsw1 is 33 amino acids, a length similar to what is found for human HP1 α but different from fission yeast Swi6, which has a long hinge region.

#### 3.2.2. Lsw1 Interacts with Itself

**Figure 5:**
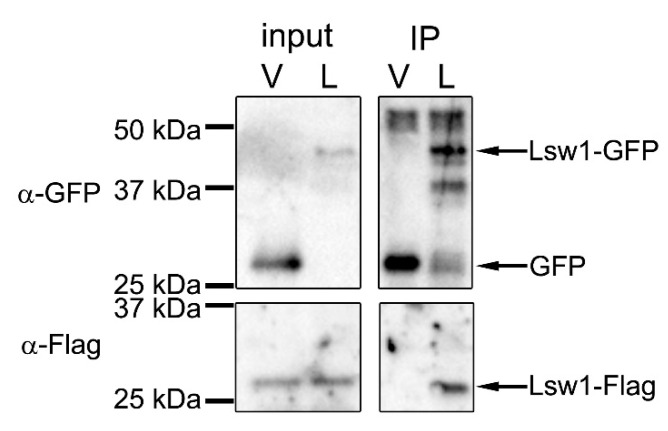
Self-interaction of Lsw1. Δ*swi6* cells (YTP1847) containing pRep42-Lsw1-Flag and either pGP4110 (V) or pGP4110-Lsw1 (L) were grown in minimal medium in the absence thiamine at 30 °C for 20 h. The cell lysates were precipitated with anti-GFP antibody, and precipitated proteins were detected by Western blotting using anti-GFP and anti-Flag M2 antibodies.

Self-interaction of Swi6 is thought to cause folding of chromatin that triggers transcriptional repression [37], such that self-interaction plays an important role in formation of heterochromatin. Swi6 interacts with itself via CD–CD or CSD–CSD binding [38]. The CD–CD interaction is mediated by the ARK-loop (Figure 4, open box) [8]. In Lsw1, the corresponding amino acids are NKK, which is very similar to ARK in which two basic amino acids line up. The CSD in Swi6 contains a dimerization motif [36], and mutation of one residue (L315E) severely compromises self-interaction [39]. This residue is conserved in Lsw1 (Figure 4, asterisk), and the surrounding amino acid residues are also conserved. These findings suggest that Lsw1 interacts with itself. To test this experimentally, co-immunoprecipitation analysis was employed. Lsw1 was isolated by RT-PCR using *L. starkeyi* cDNA, fused at the C-terminus with GFP or 3xFlag, and put under the control of the *nmt41* promoter. Lsw1-Flag was inserted into the pRep42 plasmid, which has *ura4^+^* as a selection marker. Lsw1-GFP was inserted into the pRep41 (pGP4110) plasmid, which has *LEU2* as a selection marker. Cells were transformed with the two plasmids and selected in medium lacking both uracil and leucine. The genes were regulated together with thiamine. To eliminate the effects of Swi6, Lsw1-Flag and Lsw1-GFP were overexpressed in the *swi6*^+^ gene deleted cell Δ*swi6*, and the cell lysates were immunoprecipitated with the anti-GFP antibody. It was found that Lsw1-Flag co-immunoprecipitated with Lsw1-GFP, but not with GFP alone (Figure 5, IP). The same results were obtained in independent transformants. These results strongly indicate that Lsw1 interacts with itself. However, the possibility that Lsw1 self-interaction is mediated by RNA or DNA cannot be excluded.

#### 3.2.3. Lsw1 Localizes to Heterochromatin in an H3K9 Methylation Dependent Manner

**Figure 6:**
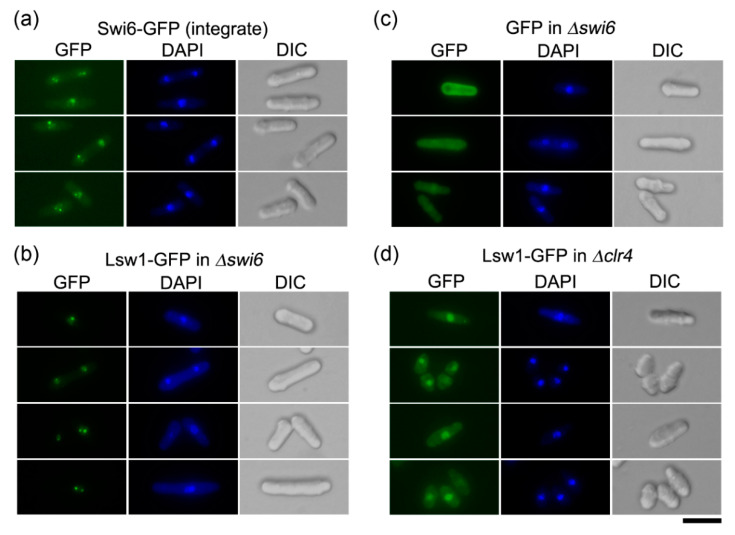
Lsw1 localizes to heterochromatin in a H3K9 methylation dependent manner. (**a**) Swi6-GFP (YTP1889) cells were grown in YES medium at 30 °C overnight, then fixed with methanol. (**b**,**c**) Δ*swi6* cells (YTP1847) containing pGP4110-Lsw1 (**b**) or pGP4110 (**c**) were grown in minimal medium supplement with uracil and without thiamine at 30 °C for 20 h, then fixed with methanol. (**d**) Δ*clr4* cells (YTP1840) containing pGP4110-Lsw1 were grown in minimal medium in the absence of thiamine at 26 °C for 20 h, then fixed with methanol. Bar, 10 μm.

Swi6 is mainly localized to heterochromatin, which clusters in 1–3 spots, corresponding to the pericentromeric region, subtelomeric region, and mating type locus [39,40]. Swi6-GFP, when expressed from a native promoter upon integration in the *swi6*^+^ locus, can be detected as 1–3 spots in a nucleus (Figure 6a). To eliminate the impact of endogenous Swi6, Lsw1-GFP was overexpressed in the *swi6* cells. The Lsw1-GFP signal localized to distinct spots, as observed for Swi6-GFP (Figure 6b). These spots could not be detected in cells overexpressing GFP alone (Figure 6c), suggesting that Lsw1 localizes to fission yeast heterochromatin regions. Swi6 specifically binds to H3K9me, and Swi6 localization is disrupted by loss of H3K9me [5]. To ascertain whether Lsw1 localization depends on H3K9me, Lsw1-GFP was overexpressed in Δ*clr4* cells, which lack Clr4, a protein that preferentially methylates H3K9 [10]. Lsw1-GFP can be detected in the nucleoplasm; however, the nuclear spots observed in Δ*swi6* cells are completely absent in Δ*clr4* cells (Figure 6d).

**Figure 7:**
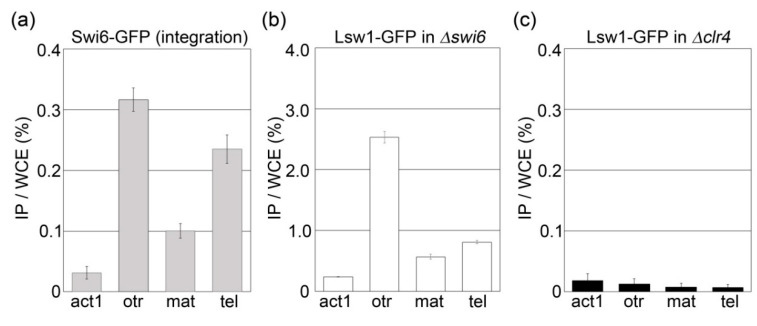
Lsw1 binds to fission yeast heterochromatin regions. Cell lysates were prepared from Swi6-GFP (YTP1889, (**a**)), Δ*swi6* (YTP1847, (**b**)) or Δ*clr4* (YTP1901, (**c**)) containing pGP4110-Lsw1. DNA co-immunoprecipitated with anti-GFP antibody was quantified by real-time PCR analysis of the outer centromere (*otr*) region, mating type locus (mat), or subtelomere (tel). *act1* was used as a negative control. The amount of immunoprecipitated DNA was calculated as a percentage of whole-cell extract (WCE). The error bars indicate the standard deviation from three or four independent experiments.

The results do not exclude the possibility that Lsw1 spots were masked by its localization to the nucleoplasm. To clarify whether Lsw1 binds heterochromatin in a H3K9 methylation dependent manner, a ChIP assay was performed. As expected, Swi6-GFP accumulated at the pericentromeric heterochromatin region (*otr*), mating type locus (mat), and subtelomere (tel) (Figure 7a). In Δ*swi6* cells overexpressing Lsw1-GFP, Lsw1-GFP primarily bound to the *otr*; accumulation at tel and mat was lower but significant as compared to accumulation at the euchromatin region *act1^+^* (Figure 7b). This accumulation at heterochromatic regions was absent in Δ*clr4* cells (Figure 7c). Consequently, it was concluded that Lsw1 binds to these three heterochromatin regions in a H3K9me-dependant manner. Most Saccharomycotina species have lost the ancestral form of heterochromatin formed via H3K9me. The authors of [2] suggested that the silencing mechanism, which is required for mating-type switching, evolved in the Saccharomycotina lineage relatively soon after the H3K9me form of heterochromatin was lost. Notably, it was found that Lsw1 binds not only to the fission yeast centromere but also to mat, and accordingly the *L. starkeyi* mating type locus might be silenced by heterochromatin.

**Figure 8:**
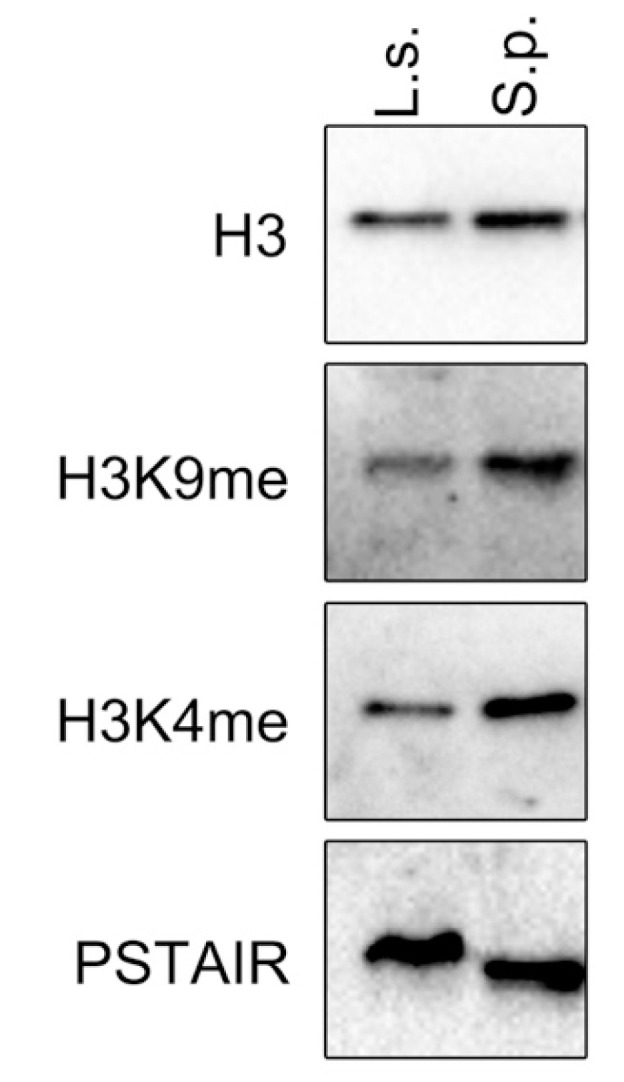
*L. starkeyi* chromatin contains H3K9me. Cell extracts prepared from *L. starkeyi* (L.s.) or *S. pombe* (S.p.) were analyzed by Western blotting. Levels of Cdc2 (anti-PSTAIR) were used as a loading control.

Methylation of histone H3 at lys4 (H3K4me) is associated with transcriptionally active chromatin. However, although H3K9me is found in *S. pombe* and many other eukaryotes, it has not been detected in *S. cerevisiae* chromatin. Next, the presence of H3K9me in *L. starkeyi* that is recognized and bound by Lsw1 was investigated. To do this, *L. starkeyi* cell lysate was analyzed by Western blot. As shown in Figure 8, methylation of histone H3 at lysine 9 was detected. The presence of H3K9me strongly suggests that Lsw1 binds to H3K9me in *L. starkeyi* cells.

## 4. Discussion

In this work, *L. starkeyi* homologs of CENP-A (CENP-A^L.s.^) and heterochromatin protein 1 (Lsw1) were identified. CENP-A^L.s.^ can replace *S. pombe* Cnp1, and Lsw1 interacts with itself and binds heterochromatin regions in an H3K9me-dependent manner. Moreover, *L. starkeyi* chromatin was found to contain methylated histone H3K9, implying that *L. starkeyi* retained a primitive heterochromatin structure that relies on H3K9me, even though that structure was lost in other Saccharomycotina.

CENP-A^L.s.^ is able to localize to the fission yeast centromere region. In fission yeast, the number of nuclei reflects the cell cycle state of the cells; during G1 and S phases, the cells are binuclear, and at the G2 phase, the cells have a single nucleus. Centromere loading of Cnp1 occurs during the G2 phase [20,41,42]. Additionally, previous reports showed that Cnp1 is able to accomplish centromere loading in the S phase, suggesting that Cnp1 loading in the S phase occurs via formation of new centromere nucleosomes during DNA replication [20,43]. As shown in Figure 3a, a centromeric CENP-A^L.s.^ signal is clearly visible in a single-nucleus cells, whereas the signal is very weak in binucleate cells. The weak CENP-A^L.s.^ signal detected in binucleate cells might be due to preferential loading of Cnp1 rather than CENP-A^L.s.^, as Cnp1 is expressed in G1 to early S phase [14,44]. In G2-phase cells, CENP-A^L.s.^ is constitutively overexpressed, whereas the cells barely express Cnp1 in this phase. Consequently, CENP-A^L.s.^ is mainly loaded on the centromere in single-nucleus cells, where its signal is clearly detected. This model is supported by the observation that when Cnp1 is ectopically expressed in phases other than the G1 phase, Cnp1 can still target the centromere, and further, detection of Cnp1 localization correlates with the strength of the promoter [44]. Centromere loading of CENP-A^L.s.^ occurs in the same way as Cnp1, and CENP-A^L.s.^ binds faithfully to the core centromere region (*cnt*), indicating that CENP-A^L.s.^ is a centromere-specific histone H3 variant protein of *L. starkeyi*.

**Figure 9:**
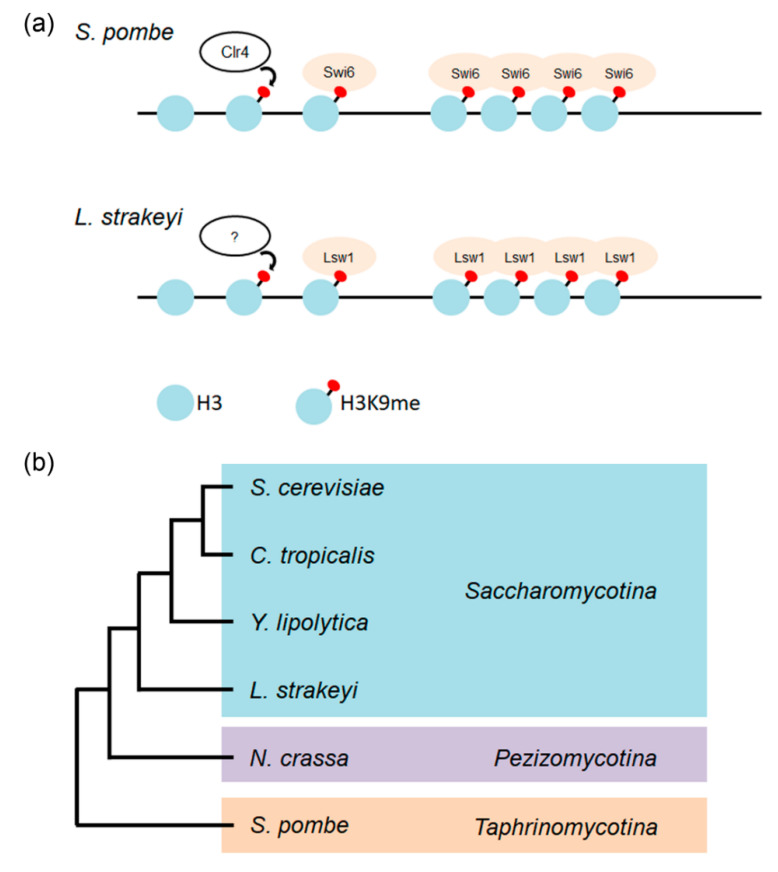
Heterochromatin formation. (**a**) Schematic of heterochromatinization of *S. pombe* (upper) and corresponding putative heterochromatinization of *L. starkeyi* (lower). (**b**) An evolutionary tree showing the relationship among yeasts. Phylogenetic tree based on [2,48].

In fission yeast, a centromeric heterochromatin structure is maintained and stabilized by the RNAi machinery [11,45]. In contrast, *S. cerevisiae* have lost essential components of the RNAi machinery. In some budding yeasts (e.g., *Saccharomyces castellii*, *Kluyveromyces polysporus,* and *C. albicans*), Argonaut and Dicer proteins, which are components of the RNAi machinery, have been found, but these species nevertheless lack a functional RNAi system [46]. In *L. starkeyi,* the RNAi machinery genes have been identified and cloned (Takayama, unpublished results). The possibility that *L. starkeyi* contains heterochromatin and RNAi machinery but does not use it cannot be excluded. The mechanism of heterochromatin assembly is similar in yeast and humans, and heterochromatin assembly and its functions have been extensively analyzed using fission yeast. H3K9 methylated by the evolutionarily conserved Clr4 protein serves as a binding site for Swi6. Swi6 molecules bound to H3K9me then induce oligomerization through Swi6 self-interaction activity. The oligomerization of Swi6 promotes condensation of chromatin, resulting in gene silencing (Figure 9a upper) [47]. The *L. starkeyi* Swi6 homolog Lsw1 binds to fission yeast heterochromatin regions in a H3K9me-dependent manner, and Lsw1 can also self-interact. Analogous to assembly of fission yeast heterochromatin, it is most likely that Lsw1 is recruited to methylated H3K9 on chromatin, and that bound Lsw1 molecules oligomerize via its self-interaction activity, resulting in formation of heterochromatin (Figure 9a lower). This strongly suggests that *L. starkeyi* is the first organism among Saccharomycotina species to be identified that forms heterochromatin via H3K9me.

Centromere organization has been reported for some yeasts [48,49,50]. For example, *S. cerevisiae* centromeres consist of a specific 125 bp of DNA with a consensus sequence consisting of two short motifs (CDE I and CDE III), and these motifs are separated by an AT-rich sequence (CDE II). The *Candida tropicalis* centromere comprises a non-repetitive core flanked by inverted repeats and has a total length of 10–17 kb, such that it is sometimes called a ‘small regional centromere’ [50]. The centromere of *Yarrowia lipolytica*, one of the most-studied Saccharomycotina species for lipid accumulation, also has a CDE I-CDEII-CDE III architecture, and its centromere length is about twice as long as that of *S. cerevisiae* [51]. Most Saccharomycotina species are composed of point or small regional centromeres, such that the oleaginous yeast *L. starkeyi* is likely to have a similar structure. By contrast, a regional centromere (or large regional centromere) organization is seen in *Neurospora crassa* (300 kb long, *Pezizomycotina*) and *S. pombe* (35–110 kb long, *Schizosaccharomyces*) [11,52]. In the centromeres of these species, core regions are flanked by extensive arrays of heterochromatin that are repetitive in sequence and transcriptionally inactive. Centromeric heterochromatin is required for establishment of Cnp1 centromere localization in fission yeast [18,35], indicating that heterochromatin plays a crucial role in regional centromere identity. If *L. starkeyi* centromeres contain heterochromatin but are point or small regional centromeres, they would comprise a novel type of centromere. Thus, *L. starkeyi* is a potentially attractive model for further study of chromatin, and these studies might help shed new light on the evolution of centromeres as it relates to heterochromatin.

## Figures and Tables

**Table 1:** Strains used in this study.

Strain Name	Genotype	Reference
HM123	*h^−^ leu1-32*	[13]
Sp525	*h^−^ leu1-32 ura4-D18 Δcnp1::ura4^+^ lys1^+^-cnp1-1*	[14]
YTP1840	*h^−^ leu1-32 clr4::hph*	This study
YTP1847	*h^+^ leu1-32 ura4-D18 Δswi6::kan*	This study
YTP1889	*h^+^ Swi6-GFP-kan*	This study
YTP1901	*h^+^ leu1-32 Δclr4::hph*	This study

**Table 2:** Primers used in this study.

Primer	Sequence	Utilization	Reference
CENP-A^L.s.^-F	5′-GATGAACTATACAAAATGCCACCGAGACGACAA-3′	CENP-A^L.s.^ ORF amplification	This study
CENP-A^L.s.^-R	5′-TTACCCGGGCCGCGGTCACCCGAGACCACCCCA-3′		This study
Lsw1-F	5′-TAAATCATATGTCGAATGCCTGACGTGGACGAG-3′	Lsw1 ORF amplification	This study
Lsw1-R	5′- TTTACTCATGCGGCCGCTAAACTGAATATGTGCCT-3′		This study
Flag-F	5′-GGCCGCATGAGTAAAGGGACTAGTGACTACAAGG-3′	Flag tag amplification	This study
Flag-R	5′-TTCCTTTTAGCGGCCTCATCTAGACTTGTCATCGT-3′		This study
act1-TF	5′-CTTTCTACAACGAGCTTCGTGTTG-3′	act1 amplification for ChIP	[17]
act1-TR	5′-GAGTCATCTTCTCACGGTTGGAT-3′		[17]
cnt22-F	5′-AAAGCAAACAGCAGTAACCTTGTAA-3′	cnt amplification for ChIP	[17]
cnt22-R	5′-TGCGTCCTTATATGCGGCTTA-3′		[17]
dg1-TF	5′-CATGGAACTACGTCAGGAGTGG-3′	otr amplification for ChIP	[17]
dg1-TR	5′-TGCCCTGTTCACTTATCTAATTCG-3′		[17]
tel-TF	5′-ACACTACTTGGCCGTTCGGTTA-3′	tel amplification for ChIP	[18]
tel-TR	5′-AGTCACCGGGAACCACAAAA-3′		[18]
matK-F	5′-TCTTCCCTGCGTTGGACTTC-3′	mat amplification for ChIP	[19]
matK-R	5′-CACCCTACCATCCGTGTTACCT-3′		[19]

## References

[1] Henikoff S., Ahmad K., Malik H.S. (2001). The centromere paradox: Stable inheritance with rapidly evolving DNA. Science.

[2] Riley R., Haridas S., Wolfe K.H., Lopes M.R., Hittinger C.T., Goker M., Salamov A.A., Wisecaver J.H., Long T.M., Calvey C.H. (2016). Comparative genomics of biotechnologically important yeasts. Proc. Natl. Acad. Sci. USA.

[3] Kobayashi N., Suzuki Y., Schoenfeld L.W., Muller C.A., Nieduszynski C., Wolfe K.H., Tanaka T.U. (2015). Discovery of an unconventional centromere in budding yeast redefines evolution of point centromeres. Curr. Biol..

[4] Takahashi K., Yamada H., Yanagida M. (1994). Fission yeast minichromosome loss mutants mis cause lethal aneuploidy and replication abnormality. Mol. Biol. Cell.

[5] Partridge J.F., Borgstrom B., Allshire R.C. (2000). Distinct protein interaction domains and protein spreading in a complex centromere. Genes Dev..

[6] Volpe T., Martienssen R.A. (2011). RNA interference and heterochromatin assembly. Cold Spring Harb. Perspect. Biol..

[7] Pidoux A.L., Allshire R.C. (2005). The role of heterochromatin in centromere function. Philos. Trans. R. Soc. Lond. Ser. B Biol. Sci..

[8] Canzio D., Liao M., Naber N., Pate E., Larson A., Wu S., Marina D.B., Garcia J.F., Madhani H.D., Cooke R. (2013). A conformational switch in HP1 releases auto-inhibition to drive heterochromatin assembly. Nature.

[9] Keller C., Adaixo R., Stunnenberg R., Woolcock K.J., Hiller S., Buhler M. (2012). HP1(Swi6) mediates the recognition and destruction of heterochromatic RNA transcripts. Mol. Cell.

[10] Nakayama J., Rice J.C., Strahl B.D., Allis C.D., Grewal S.I. (2001). Role of histone H3 lysine 9 methylation in epigenetic control of heterochromatin assembly. Science.

[11] Allshire R.C., Ekwall K. (2015). Epigenetic regulation of chromatin states in schizosaccharomyces pombe. Cold Spring Harb. Perspect. Biol..

[12] Folco H.D., Campbell C.S., May K.M., Espinoza C.A., Oegema K., Hardwick K.G., Grewal S.I.S., Desai A. (2015). The CENP-A N-tail confers epigenetic stability to centromeres via the CENP-T branch of the CCAN in fission yeast. Curr. Biol..

[13] Takayama Y., Takahashi K. (2007). Differential regulation of repeated histone genes during the fission yeast cell cycle. Nucleic Acids Res..

[14] Takahashi K., Chen E.S., Yanagida M. (2000). Requirement of Mis6 centromere connector for localizing a CENP-A-like protein in fission yeast. Science.

[15] Lyne R., Burns G., Mata J., Penkett C.J., Rustici G., Chen D., Langford C., Vetrie D., Bahler J. (2003). Whole-genome microarrays of fission yeast: Characteristics, accuracy, reproducibility, and processing of array data. BMC Genom..

[16] Forsburg S.L. (1993). Comparison of Schizosaccharomyces pombe expression systems. Nucleic Acids Res..

[17] Takayama Y., Mamnun Y.M., Trickey M., Dhut S., Masuda F., Yamano H., Toda T., Saitoh S. (2010). Hsk1- and SCF(Pof3)-dependent proteolysis of S. pombe Ams2 ensures histone homeostasis and centromere function. Dev. Cell.

[18] Ishii K., Ogiyama Y., Chikashige Y., Soejima S., Masuda F., Kakuma T., Hiraoka Y., Takahashi K. (2008). Heterochromatin integrity affects chromosome reorganization after centromere dysfunction. Science.

[19] Hayashi M.T., Takahashi T.S., Nakagawa T., Nakayama J., Masukata H. (2009). The heterochromatin protein Swi6/HP1 activates replication origins at the pericentromeric region and silent mating-type locus. Nat. Cell Biol..

[20] Takayama Y., Sato H., Saitoh S., Ogiyama Y., Masuda F., Takahashi K. (2008). Biphasic incorporation of centromeric histone CENP-A in fission yeast. Mol. Biol. Cell.

[21] Takayama Y., Shirai M., Masuda F. (2016). Characterisation of functional domains in fission yeast Ams2 that are required for core histone gene transcription. Sci. Rep..

[22] Li P.C., Chretien L., Cote J., Kelly T.J., Forsburg S.L.S. (2011). pombe replication protein Cdc18 (Cdc6) interacts with Swi6 (HP1) heterochromatin protein: Region specific effects and replication timing in the centromere. Cell Cycle (Georget. Tex.).

[23] Mamnun Y.M., Katayama S., Toda T. (2006). Fission yeast Mcl1 interacts with SCF^Pof3^ and is required for centromere formation. Biochem. Biophys. Res. Commun..

[24] Vermaak D., Hayden H.S., Henikoff S. (2002). Centromere targeting element within the histone fold domain of Cid. Mol. Cell. Biol..

[25] Fachinetti D., Folco H.D., Nechemia-Arbely Y., Valente L.P., Nguyen K., Wong A.J., Zhu Q., Holland A.J., Desai A., Jansen L.E. (2013). A two-step mechanism for epigenetic specification of centromere identity and function. Nat. Cell Biol..

[26] Sato H., Masuda F., Takayama Y., Takahashi K., Saitoh S. (2012). Epigenetic inactivation and subsequent heterochromatinization of a centromere stabilize dicentric chromosomes. Curr. Biol..

[27] Au W.C., Zhang T., Mishra P.K., Eisenstatt J.R., Walker R.L., Ocampo J., Dawson A., Warren J., Costanzo M., Baryshnikova A. (2020). Skp, Cullin, F-box (SCF)-Met30 and SCF-Cdc4-Mediated proteolysis of CENP-A prevents mislocalization of CENP-A for chromosomal stability in budding yeast. PLoS Genet..

[28] Ohkuni K., Suva E., Au W.C., Walker R.L., Levy-Myers R., Meltzer P.S., Baker R.E., Basrai M.A. (2020). Deposition of centromeric histone H3 Variant CENP-A/Cse4 into chromatin is facilitated by its C-terminal sumoylation. Genetics.

[29] Tan H.L., Lim K.K., Yang Q., Fan J.S., Sayed A.M.M., Low L.S., Ren B., Lim T.K., Lin Q., Mok Y.K. (2018). Prolyl isomerization of the CENP-A N-terminus regulates centromeric integrity in fission yeast. Nucleic Acids Res..

[30] Oguro Y., Yamazaki H., Ara S., Shida Y., Ogasawara W., Takagi M., Takaku H. (2017). Efficient gene targeting in non-homologous end-joining-deficient Lipomyces starkeyi strains. Curr. Genet..

[31] McNeil B.A., Stuart D.T. (2018). Optimization of C16 and C18 fatty alcohol production by an engineered strain of Lipomyces starkeyi. J. Ind. Microbiol. Biotechnol..

[32] Oguro Y., Yamazaki H., Shida Y., Ogasawara W., Takagi M., Takaku H. (2015). Multicopy integration and expression of heterologous genes in the oleaginous yeast, Lipomyces starkeyi. Biosci. Biotechnol. Biochem..

[33] Salunke D., Manglekar R., Gadre R., Nene S., Harsulkar A.M. (2015). Production of polyunsaturated fatty acids in recombinant Lipomyces starkeyi through submerged fermentation. Bioprocess Biosyst. Eng..

[34] Yang J., Sun S., Zhang S., Gonzalez M., Dong Q., Chi Z., Chen Y.-h., Li F. (2018). Heterochromatin and RNAi regulate centromeres by protecting CENP-A from ubiquitin-mediated degradation. PLoS Genet..

[35] Kagansky A., Folco H.D., Almeida R., Pidoux A.L., Boukaba A., Simmer F., Urano T., Hamilton G.L., Allshire R.C. (2009). Synthetic heterochromatin bypasses RNAi and centromeric repeats to establish functional centromeres. Science.

[36] Brasher S.V., Smith B.O., Fogh R.H., Nietlispach D., Thiru A., Nielsen P.R., Broadhurst R.W., Ball L.J., Murzina N.V., Laue E.D. (2000). The structure of mouse HP1 suggests a unique mode of single peptide recognition by the shadow chromo domain dimer. EMBO J..

[37] Grewal S.I., Elgin S.C. (2002). Heterochromatin: New possibilities for the inheritance of structure. Curr. Opin. Genet. Dev..

[38] Isaac R.S., Sanulli S., Tibble R., Hornsby M., Ravalin M., Craik C.S., Gross J.D., Narlikar G.J. (2017). Biochemical basis for distinct roles of the heterochromatin proteins Swi6 and Chp2. J. Mol. Biol..

[39] Haldar S., Saini A., Nanda J.S., Saini S., Singh J. (2011). Role of Swi6/HP1 self-association-mediated recruitment of Clr4/Suv39 in establishment and maintenance of heterochromatin in fission yeast. J. Biol. Chem..

[40] Ekwall K., Javerzat J.P., Lorentz A., Schmidt H., Cranston G., Allshire R. (1995). The chromodomain protein Swi6: A key component at fission yeast centromeres. Science.

[41] Lando D., Endesfelder U., Berger H., Subramanian L., Dunne P.D., McColl J., Klenerman D., Carr A.M., Sauer M., Allshire R.C. (2012). Quantitative single-molecule microscopy reveals that CENP-A(Cnp1) deposition occurs during G2 in fission yeast. Open Biol..

[42] Gonzalez M., He H., Sun S., Li C., Li F. (2013). Cell cycle-dependent deposition of CENP-A requires the Dos1/2-Cdc20 complex. Proc. Natl. Acad. Sci. USA.

[43] Takahashi K., Takayama Y., Masuda F., Kobayashi Y., Saitoh S. (2005). Two distinct pathways responsible for the loading of CENP-A to centromeres in the fission yeast cell cycle. Philos. Trans. R. Soc. Lond. Ser. B Biol. Sci..

[44] Aristizabal-Corrales D., Yang J., Li F. (2019). Cell cycle-regulated transcription of CENP-A by the MBF complex ensures optimal level of CENP-A for centromere formation. Genetics.

[45] Grewal S.I. (2010). RNAi-dependent formation of heterochromatin and its diverse functions. Curr. Opin. Genet. Dev..

[46] Drinnenberg I.A., Weinberg D.E., Xie K.T., Mower J.P., Wolfe K.H., Fink G.R., Bartel D.P. (2009). RNAi in budding yeast. Science.

[47] Zofall M., Grewal S.I. (2006). RNAi-mediated heterochromatin assembly in fission yeast. Cold Spring Harb. Symp. Quant. Biol..

[48] Dujon B.A., Louis E.J. (2017). Genome diversity and evolution in the budding yeasts (saccharomycotina). Genetics.

[49] Coughlan A.Y., Hanson S.J., Byrne K.P., Wolfe K.H. (2016). Centromeres of the yeast komagataella phaffii (pichia pastoris) have a simple inverted-repeat structure. Genome Biol. Evol..

[50] Chatterjee G., Sankaranarayanan S.R., Guin K., Thattikota Y., Padmanabhan S., Siddharthan R., Sanyal K. (2016). Repeat-Associated fission yeast-like regional centromeres in the ascomycetous budding yeast candida tropicalis. PLoS Genet..

[51] Matsuoka M. (2017). Protein binding sites on centromere DNA in the dimorphic yeast Yarrowia lipolytica. Int. Biol. Rev..

[52] Smith K.M., Phatale P.A., Sullivan C.M., Pomraning K.R., Freitag M. (2011). Heterochromatin is required for normal distribution of Neurospora crassa CenH3. Mol. Cell. Biol..

